# A FRAMEWORK FOR CONSIDERING SOCIAL AND STRUCTURAL DETERMINANTS OF HEALTH IN ALZHEIMER’S DISEASE RESEARCH AND CARE

**DOI:** 10.1093/geroni/igad104.0800

**Published:** 2023-12-21

**Authors:** Shana Stites

**Affiliations:** University of Pennsylvania, Philadelphia, Pennsylvania, United States

## Abstract

Social and structural determinants of health (SSDoH) refer to the conditions in which individuals live, work, and age. SSDoH can have cumulative effects across the life course that can have an impact on outcomes for persons with Alzheimer’s disease and Alzheimer’s disease related dementias (AD/ADRD) and their family members. In our two-part presentation, we first describe the theoretical and empirical underpinnings of core SSDoH constructs in AD/ADRD research and practice. We review empirical support for the specific SSDoH that we include and highlight their relevance in the care of older adults. The core set of SSDoH that we focus on have been identified as being important across a broad range of socially and culturally heterogeneous populations and we walk through the strengths and limits of this. We also report results from data obtained during a pilot test of these measures in the UPenn Alzheimer’s Disease Research Center (ADRC), which offer empirical support of their cultural appropriateness. We outline a rationale for the consideration of SSDoH in aging and AD/ADRD research, policy, and practice. In the second part of our presentation, we offer a case example of how sex, gender, and sexual orientation operate as core SSDoH in the healthy, aging population and in the care of older adults. In our case example, we also overview definitions, clinical considerations, and policy applications.

